# Changes in energy content of menu items at out-of-home food outlets in England after calorie labelling policy implementation: a pre–post analysis (2021–2022)

**DOI:** 10.1136/bmjph-2024-001905

**Published:** 2025-09-30

**Authors:** Michael Essman, Thomas Burgoine, Yuru Huang, Andrew Jones, Megan Polden, Eric Robinson, Stephen J Sharp, Richard Smith, Martin White, Jean Adams

**Affiliations:** 1 University of Cambridge, Cambridge, UK; 2 Liverpool John Moore’s University, Liverpool, UK; 3 University of Liverpool, Liverpool, UK; 4 Department of Public Health and Sports Science, University of Exeter, Exeter, UK

**Keywords:** Public Health, Primary Prevention, Food Services

## Abstract

**Introduction:**

Eating from out-of-home food outlets (OHFO) is common and linked to poor dietary quality, weight gain and obesity. In response, England implemented mandatory calorie labelling regulations in April 2022. The aim of this evaluation study was to examine pre–post changes in the energy content of menu items from large OHFO in England after the labelling policy.

**Methods:**

Energy content of menu items from large OHFO in England was obtained using MenuTracker, a longitudinal database of online menus. Data were collected in two waves: September 2021 (prepolicy) and September 2022 (postpolicy). Linear mixed regression models were used to estimate pre–post changes in mean energy content (kcal) for all items by food group and by chain type. We also examined reformulation by estimating energy content for removed, continuous and new items.

**Results:**

Overall, a small reduction of 9 (−2.0%) (95% CI: −16 to −1) kcal in mean energy content per item was observed postpolicy. Significant mean reductions per item were found in beverages (−36 (−16.4%); 95% CI: −52 to −21 kcal), burgers (−103 (−11.1%); 95% CI: −150 to −56 kcal) and mains (−30 (−4.2%); 95% CI: −48 to −12 kcal). By chain type, significant mean reductions per item were seen in pubs, bars and inns (−52 (−8.8%); 95% CI: −68 to −36 kcal), restaurants (−23 (−4.9%); 95% CI: −42 to −5 kcal) and sports and entertainment venues (−49 (−13.4%); 95% CI: −79 to −19 kcal). Changes were driven by the removal of higher kcal items (458 kcal, 95% CI: 394 to 523) and addition of lower kcal new items (434 kcal, 95% CI: 370 to 499). There was no significant change in energy content for continuously available items, indicating limited evidence of reformulation.

**Conclusions:**

The 2022 mandatory calorie labelling policy in England led to a small reduction in the mean energy content of menu items, primarily driven by the removal of higher calorie items and the addition of lower calorie items. Further research is needed to assess long-term effects and strategies to enhance policy impact.

**WHAT IS ALREADY KNOWN ON THIS TOPIC**

- Calorie labelling policies aim to improve public health by providing consumers with calorie information at the point of sale.
- Prior evidence, mostly from the USA, found small reductions in menu item calorie content after labelling, with limited evidence regarding which food categories were impacted most.

**WHAT THIS STUDY ADDS**

- Mandatory calorie labelling in England was associated with a 9 kcal (2%) reduction in the energy content of menu items.
- Changes were primarily due to removing higher and adding slightly lower calorie items, rather than reformulation.

**HOW THIS STUDY MIGHT AFFECT RESEARCH, PRACTICE OR POLICY**

- For the small calorie reductions observed to lead to meaningful population health improvements, consumers would need to shift purchases towards the lower-calorie items.

## Introduction

 Eating from out-of-home food outlets (OHFO) is common and associated with poorer dietary quality, weight gain and obesity, posing significant challenges to public health.^1^,^3^ A critical issue in out-of-home (OOH) eating is that individuals often underestimate their energy consumption, particularly with energy-dense foods from OHFO.^1 4 5^ A 2018 study of UK chains with 50 or more locations found 96% of meals in full-service restaurants and 70% in fast-food outlets exceeded 600 kcal—the maximum for a single meal suggested by Public Health England in 2017.^6 7^ Recognising the urgency of addressing the public health implications of OOH food consumption, the UK Government implemented mandatory calorie labelling regulations on 6 April 2022.^8^ These regulations require large OHFOs in England, defined as those with 250 or more employees, to display kilocalorie (kcal) information for menu items at the point of sale. The regulations apply to food and non-alcoholic drink items ready for immediate consumption, excluding prepackaged foods and exemptions.^9^

The evidence for the effect of energy labels on customer selections is mixed. Studies conducted in the USA present varying results, with some suggesting a reduction in daily caloric intake, while others find no significant effect on energy content ordered or consumed.^10^,^12^ In England, we found no evidence of changes in kcal purchased or consumed post-implementation.^13^ Furthermore, the type of food outlet may influence customer behaviour, with greater reductions observed in cafes and sit-down restaurants compared with fast-food outlets,^14^ potentially due to varying customer expectations. Qualitative research suggests customer expectations could be a key barrier to the impact of menu labelling on ordering lower energy items.^15^

Energy labelling policies may not only act as an information intervention to change customer behaviour but may also incentivise retailers to change the energy content of menu items.^15^ Recent qualitative work suggests that large OHFOs may take a ‘health by stealth’ approach to reformulation: gradually reducing the energy, sugar and salt content of their products as a response to labelling regulations to make food options slightly healthier without being noticed by customers.^15^ Menu changes may involve reformulating existing products, discontinuing higher energy products or adding lower energy products. A recent meta-analysis of 45 studies found that mandatory energy labelling was associated with a 15 kcal reduction per menu item,^12^ which could translate into lower intakes if consumers select lower-energy items.

Before mandatory labelling policies were implemented in the USA and England, there was some evidence that restaurants that implemented voluntary energy labelling sold lower fat and salt items than those without such labelling.^16 17^ However, this finding could reflect a preference for labelling among those selling healthier items. There is less evidence that demonstrates that mandatory labelling reduces energy content. Most of the previous evidence for national-level energy labelling policies is from the USA,^18^,^21^ where a recent study found that mandated energy labelling may have encouraged large restaurant chains to introduce lower-energy items.^22^ Another study of locally implemented labelling policies in the USA found no changes in mean energy on menus between experimental and control fast-food chains but did find locations with menu labelling offered a higher proportion of items classified as ‘healthier’ based on nutrient criteria.^23^ Although meta-analyses suggest small but potentially beneficial improvements to menus after energy labelling,^12^ customers may not benefit from average reductions if they do not select lower energy products. Therefore, it is essential to identify which food categories are most subject to change to determine where dietary improvements can be made. Reformulation feasibility is likely to vary by different food groups, which justifies analysing food group changes.

To address these research gaps, this study examines pre–post changes in the energy content of menu items before and after the calorie labelling (Out of Home Sector) (England) regulations. Our aims were to examine: (1) pre–post differences in energy content of items overall and by food group and chain type and (2) whether any changes were driven by changes in energy content of items offered or reformulation of existing items.

## Methods

Using the MenuTracker database, the first longitudinal nutritional database of food prepared out of the home in the UK,^24^ we assessed both reformulation and menu changes from before to after introduction of the labelling regulations. Reformulation was defined as changes in continuously available items at both time periods, and menu changes included removals or additions of items. We examined changes in mean kcal content of new, removed and continuously available items, as well as changes in mean kcal by food category and chain type. Additionally, we examined the proportions of menu items exceeding recommended energy intake per meal (>600 kcal) according to current guidance in England.^7^ These analyses were conducted for chains that were present in the database at both time periods (ie,‘core chains’). Finally, we conducted a full landscape analysis using all available data from all available chains at both time periods.

### Data source

Data in the longitudinal database MenuTracker were collected using web scraping techniques and PDF extraction tools from food businesses that posted calorie information for menu items online and were subject to the regulations.^24^ If available on business websites, MenuTracker collects food item name and description, serving size, energy, macronutrients, fibre, salt, allergens, special dietary information and menu section (used to determine whether items are children’s items or sharing items). We used data collected in September 2021 (prepolicy) and September 2022 (postpolicy) to minimise seasonal variation. A single scrape of data collection was done for each chain website and ran from 25 to 31 August 2021 for the September 2021 data collection and from 17 August to 3 September for the September 2022 data collection. Using data collected in September 2021 to represent the prepolicy period also allowed us to minimise the effect of early changes associated with implementation in April 2022 that might have occurred during the 6 months before the policy came into force.

MenuTracker September 2021 (preperiod) data included 79 unique OOH food businesses (henceforth referred to as chains) subject to the regulations. MenuTracker September 2022 (postperiod) included 90 chains. One chain from the preperiod did not present information online in the postperiod, and therefore, our main analysis includes the 78 chains operating at both time periods. For simplicity, these 78 chains will be referred to as core chains (online supplemental table 1). We also conducted a full landscape analysis using all available data from all chains at both time periods (ie, 79 pre and 90 post). This expanded database is described in online supplemental table 2.

### Sample size

**Figure 1:**
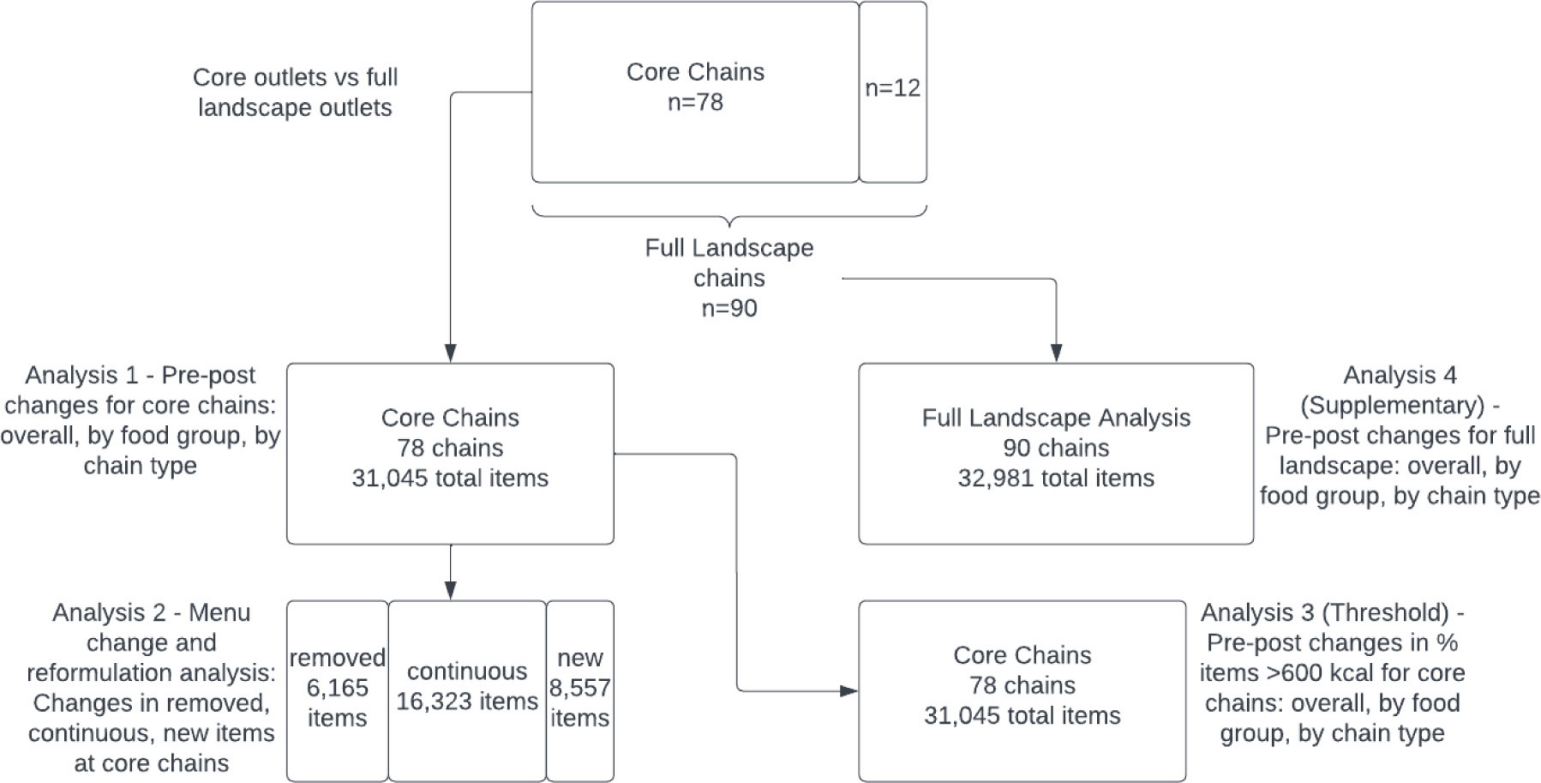
Conceptual diagram for analyses, including total number of chains and items.

The preperiod data included 19 392 menu items, reduced to 17 455 after removing duplicates. Macronutrient data were used to calculate missing kcal values where applicable (59 items). 2370 items were deleted due to having no kcal information, resulting in 15 085 unique items with kcal information. The postperiod data included 24 097 menu items. After removing 4795 duplicates and 1406 items with no kcal information, there were 17 896 unique items with kcal information remaining. Thus, our full landscape analysis consisted of a total of 32 981 items (online supplemental figure 1). When restricting the analysis to the 78 core chains, there were 15 057 items prepolicy and 15 988 items postpolicy, totalling 31 045 (figure 1).

### Food groups

Menu items were classified via a semiautomated process by ME into 12 food groups used in previous work in both the USA and UK^16 19 25^ (online supplemental table 3). Following previous work, all variations on items were included as separate items.^16^ A reliability check of food group assignments was conducted on a 2% random sample of items (n=648) with 91% agreement and coding decisions resolved between the two coders (ME and YH).

### Chain types

Chains were classified into six chain types: cafes and bakeries (henceforth referred to as cafes); Western fast food and takeaways; pubs, bars and inns; restaurants; sports and entertainment; and Asian fast food based on how chains described their outlets and food offerings on their websites. All chains are listed within each category in online supplemental tables 1 and 2.

### Removed, continuous and new items

To distinguish reformulation from other menu changes, we categorised all items at the 78 core chains into removed, continuous and new items. Continuous items were defined as items with the same name at the same outlet present in both preperiods and postperiods. We defined removed items as present in the preperiod but not the postperiod, and we defined new items as present in the postperiod but not preperiod.

Probabilistic record linkages were conducted according to published best practices, followed by manual checking, to identify items with the same name at the same period at both time points.^26 27^ Matches required the identical chain name, but item names could be a fuzzy (ie, similar but imperfect) match. Details of the linkage procedure are described in the online supplemental materials.

## Analysis

All statistical analyses were conducted in Stata V.17. We conducted four analyses, displayed in the concept map (figure 1) for clarity and described in greater detail below. These analyses were:

1. Estimate pre–post differences in mean energy (kcal) per item at core chains (n=78) overall, by food group and by chain type. This addresses aim 1 (to examine differences in energy content of items overall and by food group and chain type).
2. Estimate pre–post differences in mean energy (kcal) for each of the removed, continuous and new items at core chains (n=78) overall, by food group and by chain type. This addresses aim 2 (to examine whether any changes were driven by changes in items offered or reformulation of existing items).
3. Estimate pre–post differences in prevalence of items that exceed 600 kcal threshold at core chains (n=78) overall, by food group and by chain type. This contributes to addressing aim 1 but uses a more policy-relevant threshold, rather than the continuous metric in analysis 1.
4. Estimate pre–post differences in mean energy (kcal) using the full landscape of chains (n=90) overall, by food group and by chain type. This tests the sensitivity of analysis 1 to the chains included.

We detail the general modelling approach for analysis 1 and its adaptations for analyses 2–4. All analyses treated each menu item with equal weight, regardless of its sales volume, as we did not have access to sales data.

### Analysis 1: estimate pre–post differences in mean energy (kcal) at core chains (n=78) overall, by food group and by chain type

We used linear mixed regression models with random intercepts to estimate the mean energy content (kcal) for items overall, by food group and by chain type. Models included a three-level hierarchical structure to account for inherent clustering of data: menu items represented the first level (level 1), nested within chains (level 2), which were further nested within chain types (level 3). Models were adjusted at the item-level for children’s menu item status, sharing items and food group. Including item-level covariates adjusts for differences across outlets, as sharing platters, children’s menus and food groups differ in calorie content and size and are associated with energy content. We also conducted a sensitivity analysis that removed food group from the adjustment set, which may more closely reflect what customers see on menus. We estimated mean kcal and 95% CIs using average marginal effects, and two-way interactions between time (binary) with food group and with chain type to estimate mean kcal and 95% CIs for each combination of time and category. Pairwise comparisons of margins were conducted to assess differences between time periods for each level of the categorical variable, and each contrast is presented with 95% CIs.

### Analysis 2: estimate pre–post differences in mean energy (kcal) for each of the removed, continuous and new items at core chains (n=78) overall, by food group and by chain type

For the second analysis, we used the same modelling approach as outlined above but also included an indicator variable for whether items were new, continuous or removed. This analysis was restricted to core chains, which allows for like-for-like comparisons of changes over time within the same businesses. We estimated the marginal mean kcal and 95% CIs for removed items, new items and for continuous items. Pairwise comparisons of margins were conducted to assess differences for removed vs continuous, new versus continuous and new versus removed. We interpreted the pre–post changes in continuous items as an analysis of reformulation.

### Analysis 3 (threshold): estimate pre–post differences in prevalence of items that exceed 600 kcal threshold at core chains (n=78) overall, by food group, by chain type and by new, continuous and removed

Our third analysis examined the proportion of items that exceeded England’s per meal recommendations (>600 kcal) before and after the policy using the same three-level hierarchical structure but in this case mixed effects logistic regression models for the binary outcome (25). We estimated the marginal probability of this outcome (>600 kcal) by overall menu items, food group and chain type at each time period.

### Analysis 4 (supplementary): estimate pre–post differences in mean energy (kcal) using the full landscape of chains (n=90) overall, by food group and by chain type

The final analysis was a full landscape analysis using all available data, which includes all items from chains that are included in MenuTracker and posted calorie information online either prepolicy (n=79) or postpolicy (n=90). We followed the same modelling approach as the first analysis. We also estimated the same two-way interaction marginal effects for each food type over time and each chain type over time.

## Results

### Descriptive statistics for item availability at each data collection wave

Descriptions of item availability in each data collection wave (prepolicy September 2021 and postpolicy September 2022) at core chains and full landscape chains are presented in table 1. There were approximately 2000 more menu items present in the full landscape analysis than the core chain analysis (table 1). The most common food groups at both prepolicy and postpolicy were beverages and mains, and the least common items were soup and fried potatoes (table 1). The number of items at each chain as well as the chain type classifications is presented in online supplemental tables 1 and 2.

**Table 1:** Summary statistics for item availability at core chains (n=78) and full landscape chains (n=90)

	Core chains		Full landscape	
	Prepolicy (September 2021)	Postpolicy (September 2022)	Prepolicy (September 2021)	Postpolicy (September 2022)
Chains, n	78	78	79	90
Menu items, n	15 057	15 988	15 085	17 896
Menu items by food group, n (%)				
Appetisers and sides	1869 (12.4)	2409 (15.1)	1869 (12.4)	2824 (15.8)
Baked goods	397 (2.6)	451 (2.8)	410 (2.7)	519 (2.9)
Beverages	3474 (23.1)	4110 (25.7)	3474 (23.0)	4454 (24.9)
Burgers	411 (2.7)	388 (2.4)	411 (2.7)	490 (2.7)
Desserts	1164 (7.7)	1493 (9.3)	1171 (7.8)	1642 (9.2)
Fried potatoes	222 (1.5)	202 (1.3)	222 (1.5)	228 (1.3)
Mains	3427 (22.8)	2926 (18.3)	3428 (22.7)	3461 (19.3)
Pizza	2093 (13.9)	2008 (12.6)	2086 (13.8)	2044 (11.4)
Salads	338 (2.2)	439 (2.8)	338 (2.2)	494 (2.8)
Sandwiches	796 (5.3)	905 (5.7)	801 (5.3)	1015 (5.7)
Soup	102 (0.7)	110 (0.7)	102 (0.7)	133 (0.7)
Toppings and ingredients	764 (5.1)	547 (3.4)	771 (5.1)	587 (3.3)
Menu items by chain type, n (%)				
Cafes	3792 (25.2)	3558 (22.3)	3792 (25.1)	3861 (21.6)
Fast food and takeaway	4136 (27.5)	3813 (23.9)	4164 (27.6)	3879 (21.7)
Pubs, bars and inns	3584 (23.8)	4071 (25.5)	3584 (23.8)	4735 (26.5)
Restaurants	2412 (16.0)	3045 (19.1)	2412 (16.0)	3830 (21.4)
Sport and entertainment	901 (6.0)	1164 (7.3)	901 (6.0)	1254 (7.0)
Asian fast food	232 (1.5)	337 (2.1)	232 (1.5)	337 (1.9)

### Analysis 1: estimate pre–post differences in mean energy (kcal) at core chains (n=78) overall, by food group and by chain type

**Figure 2:**
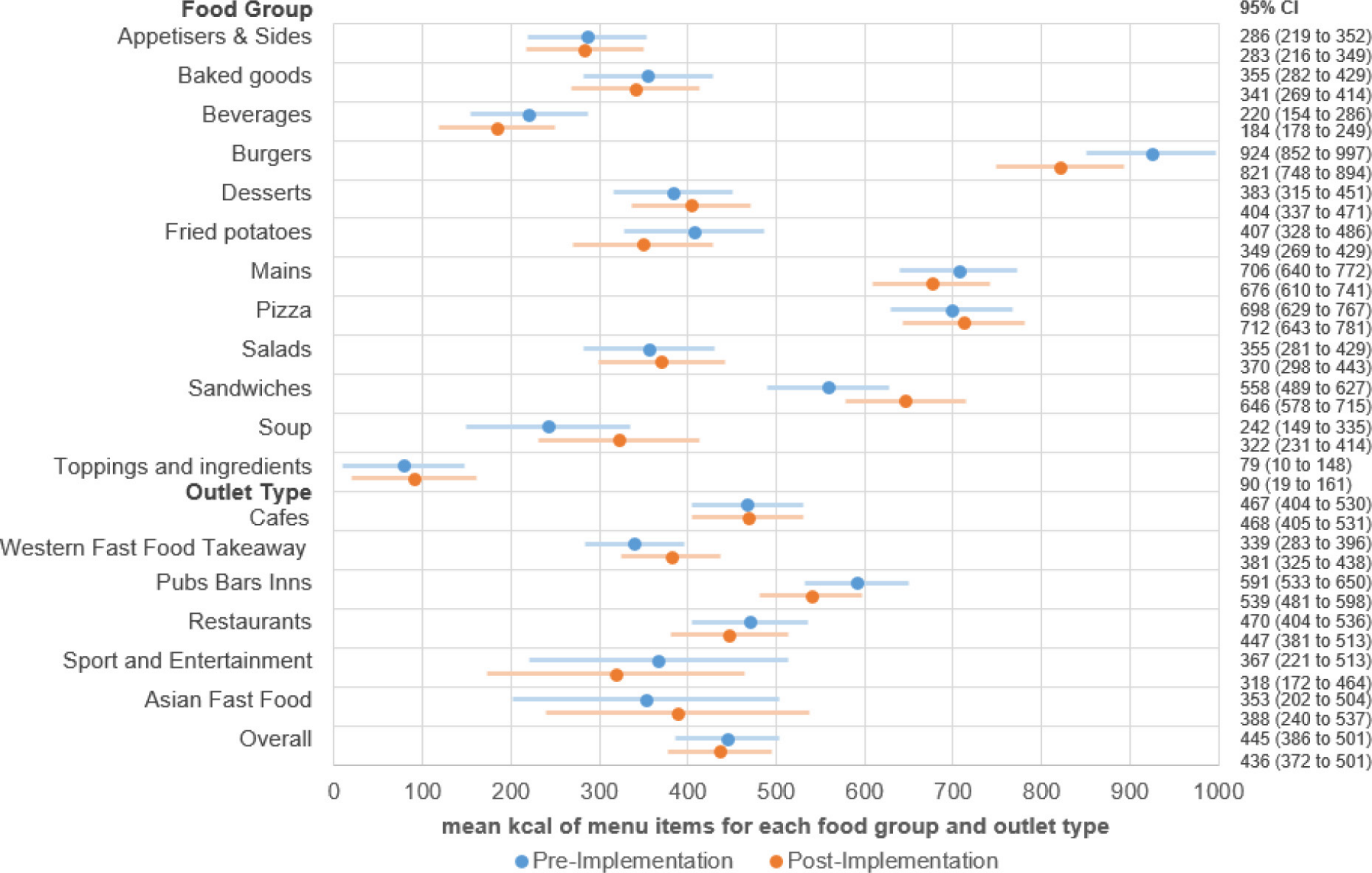
Marginal mean kcal from linear mixed model overall, by food group and by chain type for all items available at core chains (n=78) using MenuTracker data from prepolicy (September 2021) and postpolicy (September 2022), total n items=31 045.

**Figure 3:**
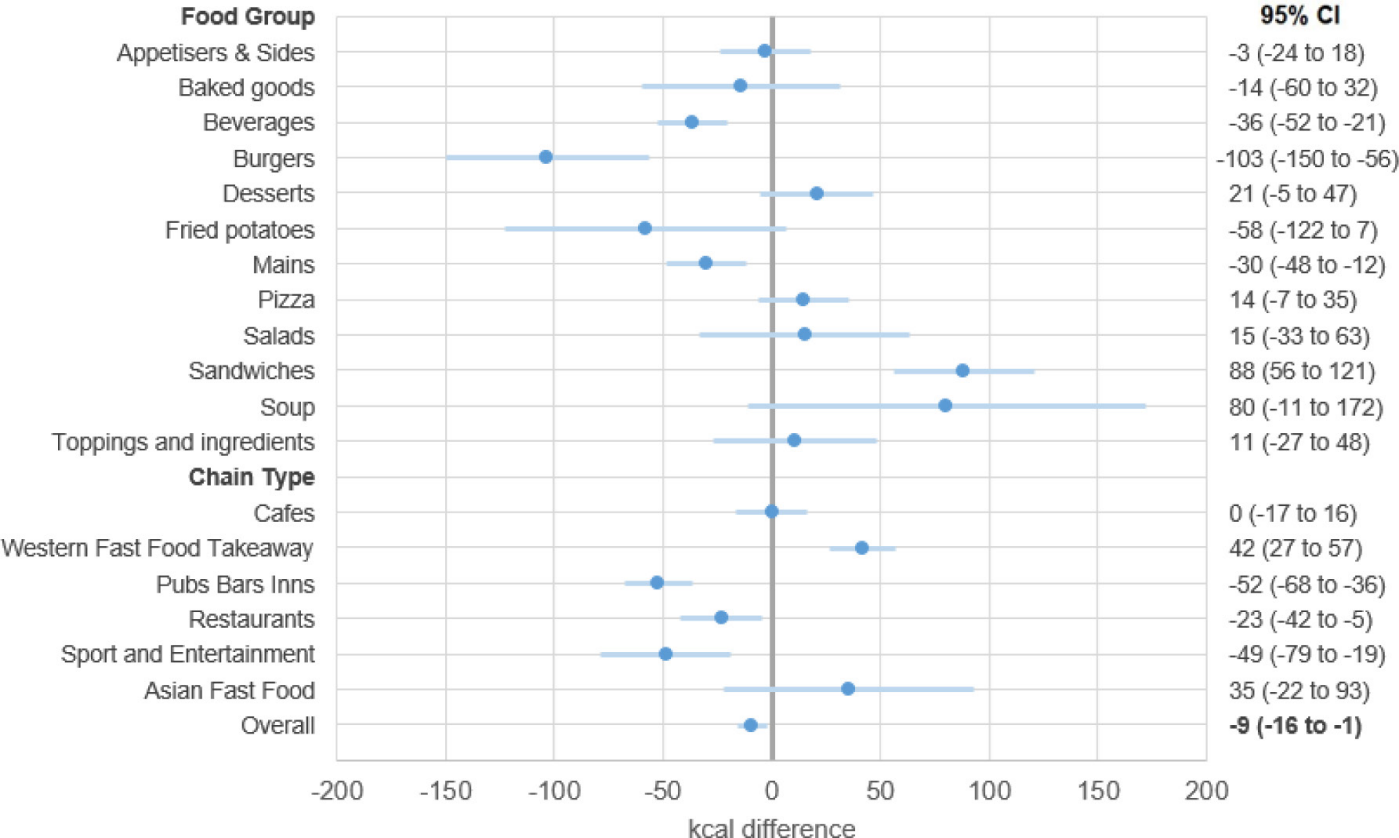
Pre–post differences in kcal overall, by food group and by chain type estimated from linear mixed model for all items available at core chains (n=78) using MenuTracker data from prepolicy (September 2021) and postpolicy (September 2022), total n items=31 045.

Figure 2 presents mean kcal content for items at core chains, both before and after the implementation of the policy, overall, by food group and by chain type, and figure 3 presents pre–post differences for items at core chains overall, by food group and by chain type. Prior to the policy, the estimated mean kcal for all items was 445 kcal, and following the policy, the estimated mean kcal decreased to 436 kcal, a 9 (–2.0%) (95% CI: −16 to −1) kcal reduction (figure 2). According to the sensitivity analysis that did not adjust for food group, overall mean kcal was 446 (95% CI 357 to 535) in the prepolicy period and 424 (95% CI 336 to 513) in the postpolicy period, a difference of 21 kcal (95% CI −30 to −12).

The highest kcal per item food groups were burgers, mains and pizzas, and the highest kcal per item chain types were restaurants, and pubs, bars and inns (figure 2). After the policy, the largest significant reductions were −103 (−11.1%) (95% CI: −150 to −56) kcal for burgers, −36 (−16.4%) (95% CI: −52 to −21) kcal for beverages and −30 (−4.2%) (95% CI: −48 to −12) kcal for mains (figure 3). Few soups were available, resulting in wide CIs for changes (figure 3). Sandwiches increased by 88 (15.8%) (95% CI: 56 to 121) kcal pre–post (figure 3). When analysed by chain type, statistically significant results included a reduction of −52 (−8.8%) (95% CI: −68 to −36) kcal at pubs, bars and inns, a reduction of −23 (−4.9%) (95% CI: −42 to −5) kcal for restaurant items, and a reduction of −49 (−13.4%) (95% CI: −79 to −19) kcal for Sports and Entertainment items. There was an increase of 42 (11.0%) (95% CI: 27 to 57) kcal for Western Fast Food and Takeaway items (figure 3).

### Analysis 2: estimate pre–post differences in mean energy (kcal) for removed, continuous and new items at core chains (n=78) overall, by food group and by chain type

**Figure 4:**
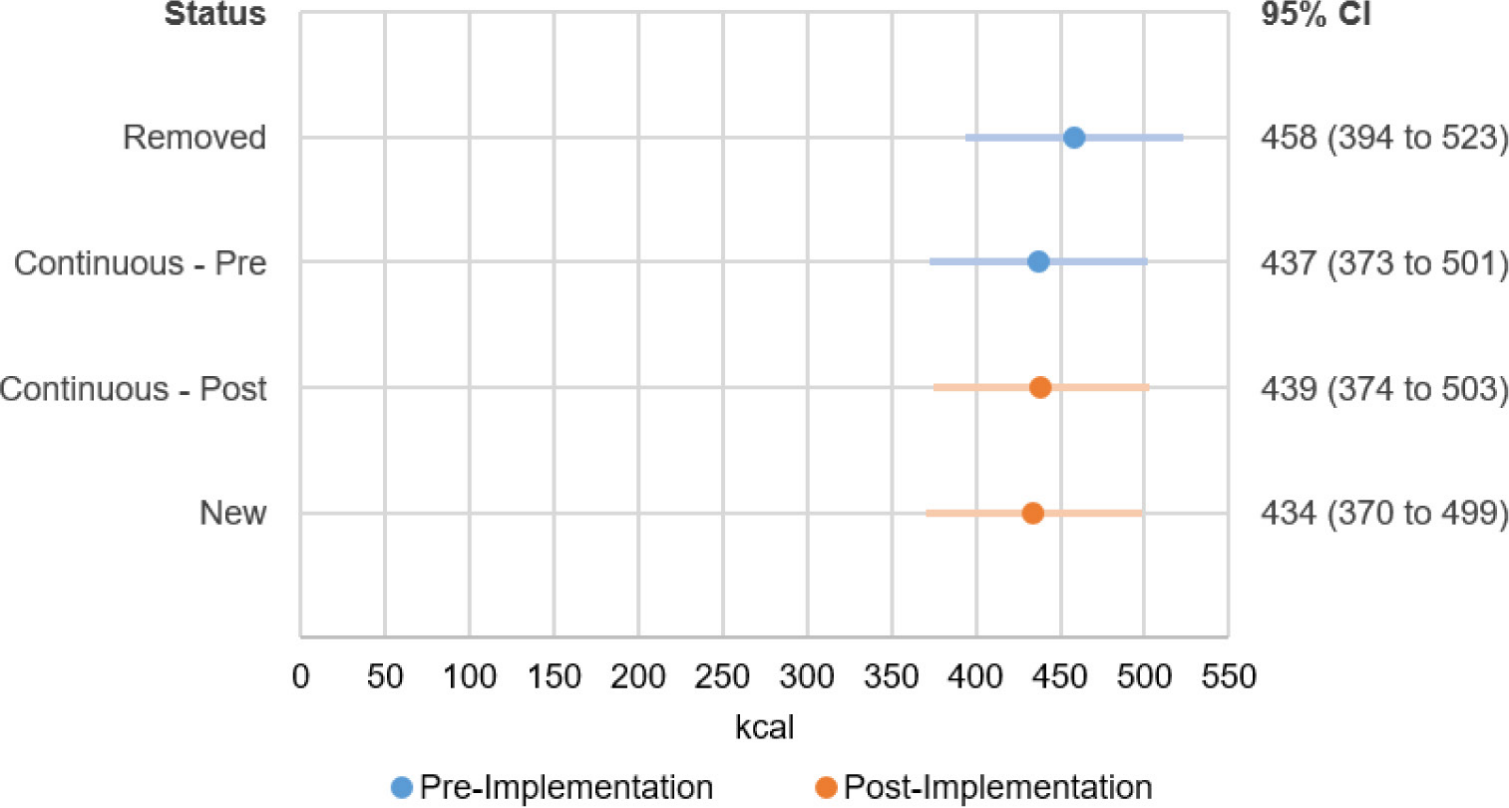
Marginal mean kcal for removed, continuous and new items estimated from linear mixed model at core chains (n=78) using MenuTracker data from prepolicy (September 2021) and postpolicy (September 2022), total n items=31 045.

Figure 4 presents estimated mean kcal content from the linear mixed model for items categorised as removed, new and continuously available, before and after the policy implementation. Prior to the policy, removed items had an estimated mean energy content of 458 (95% CI: 394 to 523) kcal, and continuously available items had 437 (95% CI: 373 to 501) kcal. After the policy, new items had 434 (95% CI: 370 to 499) kcal, and continuously available items had 439 (95% CI: 374 to 503) kcal, no change compared with prepolicy (figure 4). Removed items contained 21 (95% CI: 8 to 34) more kcal than continuous items and 25 (95% CI: 9 to 41) more kcal than new items (online supplemental figure 2).

### Analysis 3 (threshold): estimate pre–post differences in prevalence of items that exceed 600 kcal threshold at core chains (n=78) overall, by food group, by chain type and by new, continuous and removed

Online supplemental figure 3 presents the proportion of items over the 600-kcal threshold for core chains, both before and after the implementation of the policy. Prior to the policy, 21.8% of items (95% CI: 15.3% to 28.3%) were over 600 kcal, and after the policy, 22.2% of items (95% CI: 15.6% to 28.7%) were over 600 kcal, with no difference between prepolicy and postpolicy.

The food groups with the most items over 600 kcal were burgers, mains and pizzas, and the chain types with the most items over 600 kcal were restaurants and pubs, bars and inns (online supplemental figure 3). After the policy, the proportion of items exceeding 600 kcal was 3.2% (95% CI: −5.6 to −0.8) lower for beverages, 9.5% (95% CI: −15.6% to −3.4%) lower for burgers and 3.4% (95% CI: −5.6% to −1.3%) lower for mains. The proportion of items exceeding 600 kcal increased by 9.0% (95% CI 6.4% to 11.6%) for pizzas (online supplemental figure 4). When analysed by chain type, the proportion of items exceeding 600 kcal was 3.9% (95% CI: −5.7% to −2.2%) lower at pubs, bars and inns (online supplemental figure 4).

### Analysis 4: estimate pre–post differences in mean energy (kcal) using the full landscape of chains (n=90) overall, by food group and by chain type

The results from the full landscape analysis are presented in online supplemental table 4. The overall mean kcal was 458 (95% CI 412 to 505) in the prepolicy period and 450 (404 to 497) in the postpolicy period, and a difference of 8 fewer kcal (−8 to 0.2) in the postperiod that was not statistically significant. The overall pattern of results reflected analysis 1, with lower kcal values in beverages, burgers and mains, lower kcal values at restaurants and pubs, bars and inns, and an increase in kcal offered by Western fast-food chains.

## Discussion

### Statement of principal findings

To our knowledge, this study is the first to examine menu changes at OOH food chains following England’s 2022 calorie labelling policy. We found a small reduction in mean kcal after implementation of the calorie labelling policy compared with before. These mean changes were driven by the removal of higher calorie items on menus. There were no pre–post changes in kcal for continuously available items. Thus, we find some evidence for changes in menus but limited evidence for reformulation. The greatest changes in food groups were for burgers, beverages and mains. When analysed by chain type, the significant changes included reductions in kcal of items at pubs, bars and inns, restaurants and entertainment chains, and an increase at Western fast food and takeaway chains. The food groups with the most items over 600 kcal were burgers and pizzas, and the chain types with the most items over 600 kcal were restaurants and pubs, bars and inns. The full landscape analysis found a similar pattern of results to the core chain analysis.

### Interpretation of findings

The small reduction in average kcal of items available on menus we found is likely to have modest to limited impact on population health, consistent with a recent study of approximately 3000 customers that found no change in kcal purchased or consumed.^13^ Chains may have reformulated some items between the policy announcement in July 2021 and the initial data collection for this study, in September 2021.

We identified greater reductions in kcal among food groups and chain types with higher baseline kcals on menus. Changing larger kcal items may be easier due to a larger baseline portion size or high calorie density, and reducing fat content of high-calorie items could reduce energy density.^28^ Another explanation for the reductions in larger food groups could be that there is some potential embarrassment in having extremely high kcal items on menus once labelling is mandatory.^15^ Calorie reductions may be easier for beverages, where low or zero calorie options are more feasible. To account for differences in baseline kcal across item types and to improve interpretability, key results are presented as per cent differences alongside absolute differences.

We found more evidence of menu change rather than reformulation with items removed from menus being higher energy than continuous items. Thus, the impact of a calorie labelling policy on food may differ from other policies like the Soft Drinks Industry Levy, which created an economic incentive for, and was associated with substantial, reformulation.^29^ If food industry actors successfully market OOH eating as a treat where customers should indulge in high-calorie options, then business may not be incentivised to reduce the kcal of items offered.^15^ Evidence from focus groups in Ireland found that OOH eating is commonly perceived by families as a treat, with health considered a lower priority.^30^

Models not adjusted for food group changes, which indicate what customers may perceive on menus, showed a greater difference than adjusted findings. The greater reduction in mean kcal when not adjusting for food group (difference of −21 kcal, 95% CI −30 to −12) suggests that the fully adjusted model (difference of −9 kcal, 95% CI −16 to −1) may underestimate the overall effect of the calorie labelling policy. By controlling for food group, our analysis accounts for differences in the types of food items offered before and after the policy implementation. A postpolicy shift towards offering more low-calorie food groups could explain the smaller effect observed in the adjusted model. This suggests that businesses might be strategically altering their menu offerings by including more lower-calorie items. Although the results from this study demonstrate changes in items offered on menus, without linking these menu items to sales or dietary intake data, we cannot determine whether these changes in menu composition translated into changes in consumer intake or improvements in public health outcomes.

### Comparison to previous literature

Our observed overall reduction of 9 kcal aligns with a recent meta-analysis that found mandatory calorie disclosure was associated with a 15 kcal reduction in menu items.^12^ Similar to our reformulation analysis, more recent work from the USA also found no significant pre–post changes in continuously available items but observed some small reductions in mean kcal due to new items having lower kcal.^22^ Our study did identify lower mean kcal from new items, but these differences were only significant compared with removed items, not compared with continuous items. Items were similar in total kcal: 445 kcal in the preperiod and 436 kcal in the postperiod in our study, and 399 kcal in the preperiod and 388 kcal in the postperiod in the USA study.^22^ Our analysis separates foods into more categories (12 groups instead of 5), allowing for more detailed analysis of where changes occurred. Other studies, including from Ontario, Canada and King County, Washington, also reported minimal changes overall but larger reductions in full-service or sit-down restaurants.^21 31^

### Policy implications

Overall, we found limited evidence that mandatory calorie labelling in England was associated with significant changes in menu items. Alongside prior findings of no pre–post changes in kcal purchased or consumed,^13^ this suggests that while the policy’s immediate impact may be modest, even small changes can be meaningful at a population level. The limited impact observed may be related to less than perfect implementation, with only 80% of outlets displaying any calorie labelling postpolicy and only 15% meeting all implementation requirements^32^ along with low intention from local authorities to proactively check implementation in chains.^15^

Previous qualitative work identified that large OHFOs within scope of the policy were hesitant to reduce portion size due to concerns around customer satisfaction.^15^ Other strategies could support customer decisions to select lower calorie options, including more actionable contextual information beyond adults’ daily caloric needs. For example, interpretive labels are more effective than information-based labels in grocery stores.^33^ Other strategies such as price adjustments, strategic marketing or limiting total energy content of items may be warranted.

### Strengths and limitations

Our study has several strengths, including the use of the most comprehensive data available on energy content of menu items served by the OOH food sector in England that also allows for comparisons of the same chains and food items at both time points. Identifying removed, new and continuously available items, and examining changes by food and chain type, allowed us to account for systematic differences in how chains present their items. This large natural experiment examining real-world changes in a diverse set of large OHFOs may also be generalisable to other countries with similar food environments.

However, our study also has limitations. MenuTracker only includes menu information from chains that posted kcal information online before and after the policy, thus limiting how representative our findings are of the English OOH food environment. In November 2021, 256 chains were estimated to fall within scope of the regulations.^24^ We, therefore, expect our sample of 78 core chains to cover 30% of the large chain OOH food sector that posted nutrition information online at the time of data collection. Given the largest chains with the highest market share are likely to have the most resources to post kcal information online, our data may represent a larger proportion of actual OOH food sales.

Several limitations relate to the data structure. Our food group categorisations reflect how items were presented online, which varies by chain. Some chains present mixed dishes as a single item—for example, fish and chips with peas, categorised as a main—whereas others present fish, chips and peas separately, categorised as a main, fried potatoes and a side. This difference may partly explain why restaurants and pubs had higher kcal items. We adjusted for clustering at the chain and chain type level to account for these variations in data structure.

Although we assume posted kcal values are accurate, the calorie labelling regulations allow for kcal information to be within ±20% and allow several different methods for estimating energy content.^9^ In a USA-based restaurant food study, nearly one-fifth of the sample contained over 100 kcal more than the stated energy values. However, most energy and nutrient values were consistent with laboratory measurements.^34^ Any systematic changes in the accuracy of calorie estimates, or method of calculating these, over time may also have impacted our findings.

The uncontrolled before-and-after study design poses a challenge to attributing changes solely to the intervention if there was an ongoing trend in kcal information not related to the policy. However, previous work using MenuTracker data found energy content remained constant from 2018 to 2020.^25^ Some businesses may have reformulated before the policy, but they are not included if they lacked prepolicy kcal data.

We were unable to weight analyses according to the sales volume of menu items. As a result, all items were treated equally in the estimation of pre–post changes. While this approach captures changes in the energy content of items offered, it does not reflect how those changes translate to actual consumption. This limitation is particularly relevant given that, in other work, we found no statistically significant changes in energy purchased or consumed by customers between before and after policy implementation.^13^

### Recommendations for future research

Further research is needed to determine whether there will be greater long-term changes in kcal available at OHFO via gradual reductions. Future evaluations of this and similar policies in England may benefit from better surveillance data, longer time series for causal attribution and linkages to purchase data. Future research is also needed to understand whether and how consumer purchases align with the changes in menu items identified in this study. From a policy perspective, additional strategies may be needed to ensure that lower-calorie options are not only available but also selected by consumers.

## Conclusions

This study found a 9 kcal (−2.0%) reduction per item following mandatory calorie labelling in England. When not adjusting for food group, a larger reduction was observed, suggesting the change is partly influenced by differences in food group distribution. Changes were primarily driven by removing higher-calorie items rather than reformulation. When analysed by food group, the most significant reductions occurred in beverages, burgers and mains, indicating that policy impact could improve if customers select lower kcal items in these categories.

## Supplementary material

10.1136/bmjph-2024-001905 online supplemental file 1

## Data Availability

Data may be obtained from a third party and are not publicly available.
