# Supplementary material for: Changes in energy content of menu items at out-of-home food outlets in England after calorie labelling policy implementation: a pre–post analysis (2021–2022)
Source: BMJ Public Health. 2025 Sep 30;3(2):e001905. doi: 10.1136/bmjph-2024-001905 (PMC12506044; doi:10.1136/bmjph-2024-001905)
Supplement: online supplemental file 1 [file bmjph-3-2-s001.pdf]

Supplementary Methods: record linkage procedure

We used the ‘reclink’ package in Stata to facilitate probabilistic record linkage. The `reclink` command uses matching algorithms to determine the likelihood of a match between records based on the similarity of item names. This algorithmic approach was followed with manual checking by ME. There were two stages of manual checks. The first step was to check for false positive matches using the clrevmatch reviewing tool, which juxtaposes all potential matches next to each other and allows the user to confirm whether it is a correct match. An example of a potential false positive that can occur during probabilistic matching under conditions of high similarity would be an erroneous match between “Caramel Macchiato Coconut Milk 12 oz” and “Caramel Macchiato Coconut Milk 16 oz” despite only a single character difference. Thus, highly similar, but not exact, matches were manually checked to ensure they were true matches. The second manual check was to identify false negative matches that were missed during probabilistic matching. ME manually checked the entire database sorted first by chain name and then by item name to identify potential matches that were not captured by the fuzzy match. These cases occurred if the outlet changed the way the item name was recorded between pre- and post-periods, even if it was clearly the same item. For example, despite less character similarity than the macchiato example, “Beetroot Latte with coconut” is a match with “Beetroot Latte coconut milk”.

A further manual check was conducted to explore reliability of the new, continuous, and removed designations. A random chain was selected from each chain type to account for possible menu differences, resulting in 2,471 items from a total of 32,981 (7.5%). There was high agreement between the coders (96.2%).

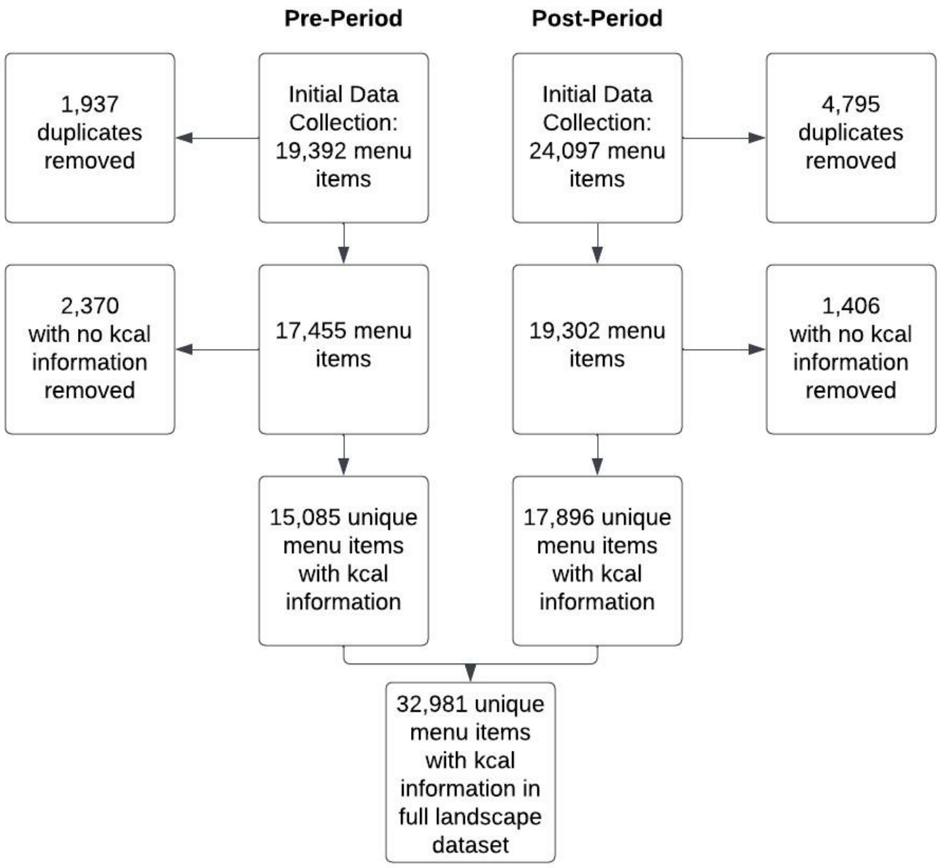

Supplementary Figure 1. Flowchart for data collection for full landscape analysis: all 90 available chains included.

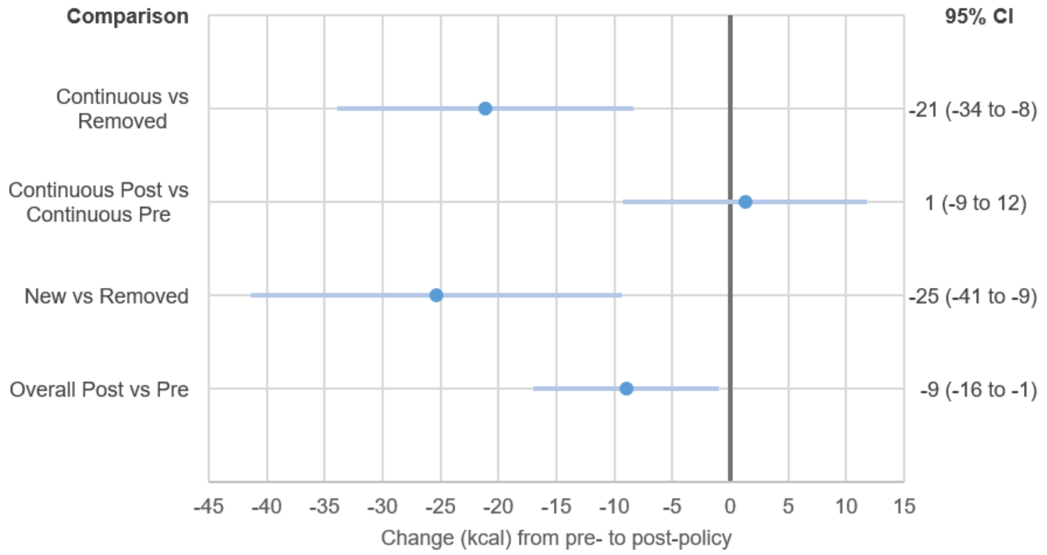

Supplementary Figure 2. Differences in kcal for comparisons of removed, new, and continuous items estimated from linear mixed model at core chains (n=78) using MenuTracker data from pre- (September 2021) and post-policy (September 2022), total n items=31,045

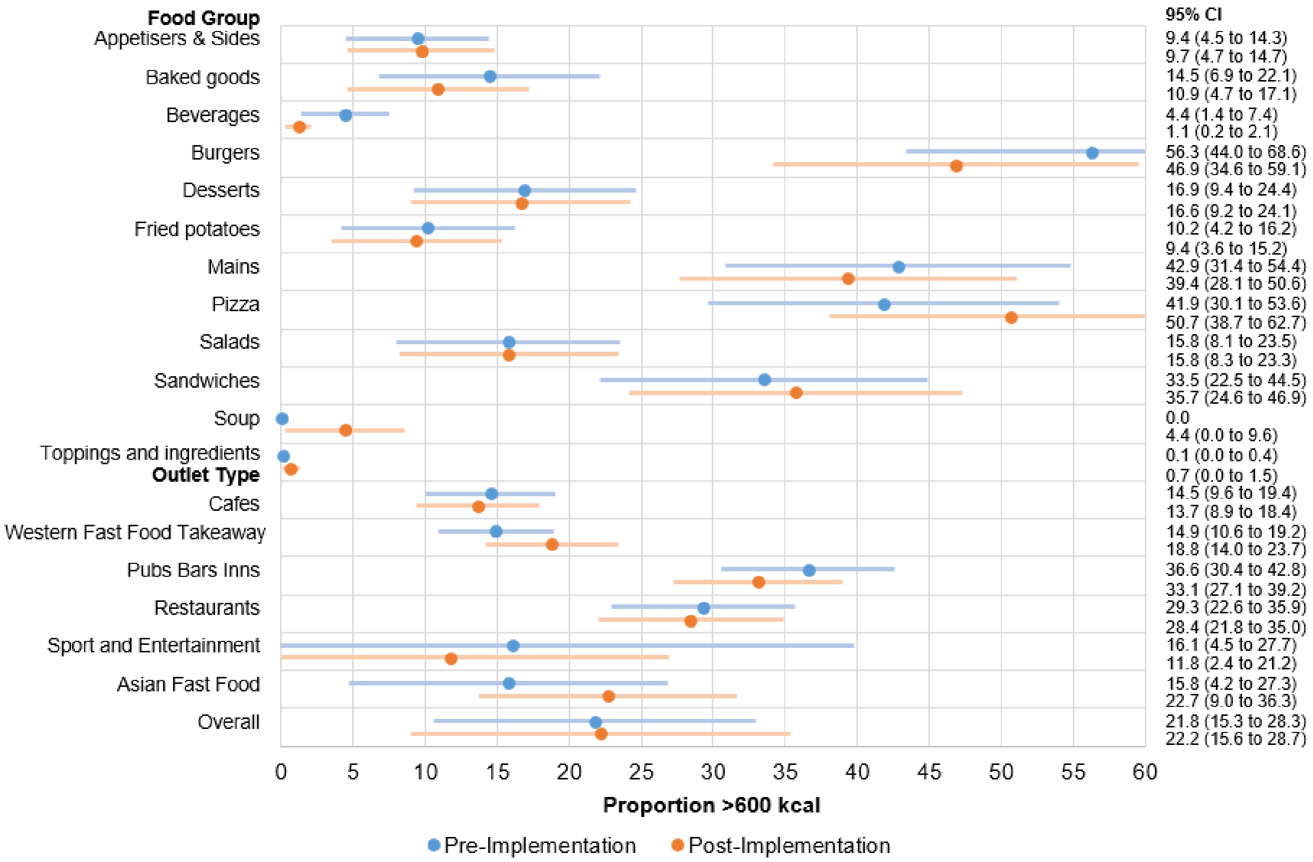

Supplementary Figure 3. Proportion of items that exceed 600 kcal estimated from linear mixed model overall, by food group, and by restaurant type for all items available at core chains (n=78) using MenuTracker data from pre- (September 2021) and post-policy (September 2022), total n items=31,045

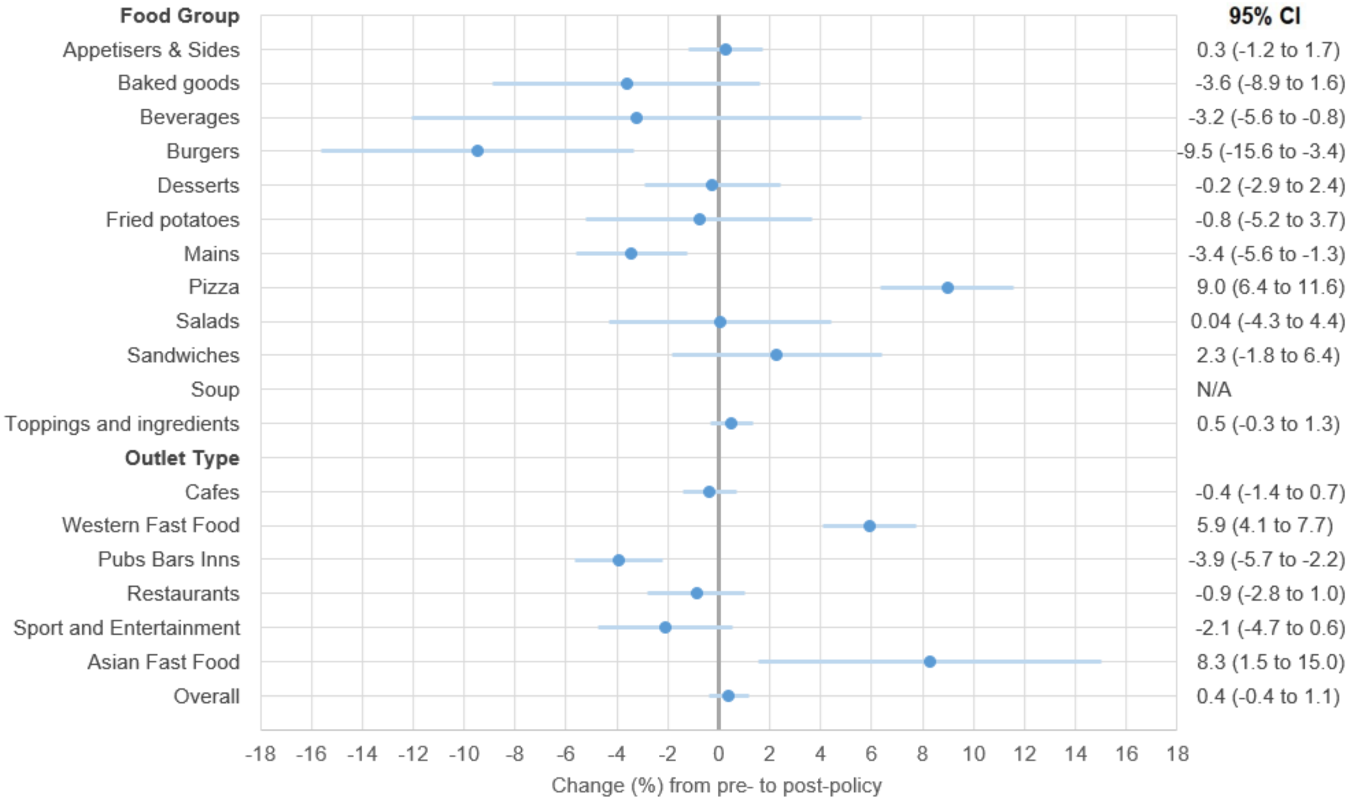

Supplementary Figure 4. Pre-post differences in proportion of items that exceed 600 kcal estimated from linear mixed model overall, by food group, and by chain type for all items available at core chains (n=78) using MenuTracker data from pre- (September 2021) and post-policy (September 2022), total n items=31,045

Supplementary Table 1. Summary statistics (n, %) for each of the 78 core chains categorized by chain type

**Chain type**	**n items**	% **of total**
**Cafes and bakeries**	**7350**	**23.7**
Boswell	684	2.2
Benugo Cafe	187	0.6
Boost Juice Bars	107	0.3
Cafe Nero	502	1.6
Coffee #1	966	3.1
Costa Coffee	1383	4.5
Greggs	221	0.7
Joe & The Juice	126	0.4
PAUL	91	0.3
Pret A Manger	364	1.2
Soho Coffee	453	1.5
Starbucks	1784	5.7
Tesco Cafe	53	0.2
The Cornish Bakery	25	0.1
Thomas the Baker	45	0.1
Tim Hortons	201	0.6
**Western Fast Food & Takeaway**	**7949**	**25.6**
Barburrito	129	0.4
Ben & Jerry's	66	0.2
Burger King	108	0.3
Coco Di Mama	1132	3.6
Crussh	285	0.9
Domino's Pizza	2436	7.8
FIVE GUYS	74	0.2
KFC	298	1.0
Krispy Kreme	50	0.2
Leon	134	0.4
McDonalds	300	1.0
Papa John's	517	1.7
Pieminister	37	0.1
Pizza Hut	598	1.9
PizzaExpress	447	1.4
Pure.	275	0.9
Subway	169	0.5
Taco Bell	328	1.1
Tortilla	27	0.1
Tossed	115	0.4
Wimpy	424	1.4
**Pubs Bars Inns**	**7655**	**24.7**

All Bar One	416	1.3
Brewhouse and Kitchen	185	0.6
Brewers Fayre	496	1.6
Chef and Brewer	402	1.3
Common Room	284	0.9
Cookhouse & Pub	431	1.4
Ember Inns	555	1.8
Farmhouse Inns	470	1.5
Flaming Grill Pub Co.	524	1.7
Hungry Horse	555	1.8
Marstons	416	1.3
Revolution Vodka Bars	289	0.9
Sizzling Pubs	745	2.4
Tank and Paddle	172	0.6
Town, Pub & Kitchen	315	1.0
Vintage Inns	497	1.6
Walkabout	177	0.6
Wetherspoon	441	1.4
Yate's	285	0.9
**Restaurants**	**5457**	**17.6**
Ask	220	0.7
Beefeater Grill	470	1.5
Bella Italia	394	1.3
Cafe Rouge	304	1.0
GBK	346	1.1
Harvester	813	2.6
Loch Fyne	219	0.7
Nandos	269	0.9
Pho	187	0.6
Stonehouse Pizza & Carvery	592	1.9
Table Table	497	1.6
The Real Greek	71	0.2
Toby Carvery	560	1.8
Wagamama	277	0.9
Zizzi	238	0.8
**Sport and Entertainment**	**2065**	**6.7**
Cineworld	524	1.7
ODEON	1134	3.7
Vue Entertainment	407	1.3
**Asian Fast Food**	**569**	**1.8**
Itsu	145	0.5
Wasabi	201	0.6
YO! Sushi	223	0.7
**Total**	**31045**	**100.0**

Supplementary Table 2. Summary statistics (n, %) for each of the 90 full landscape chains categorized by chain type

Category	n items	% of total
**Cafes and Bakeries**	**7653**	**23.2**
AMT Coffee	38	0.1
Asda Cafe	86	0.3
BOSWELL	684	2.1
Benugo Cafe	187	0.6
Boost Juice Bars	107	0.3
Caffe Nero	502	1.5
Coffee #1	966	2.9
Costa Coffee	1,383	4.2
Greggs	221	0.7
JOE & THE JUICE	126	0.4
Morrisons Cafe	179	0.5
PAUL	91	0.3
Pret A Manger	364	1.1
Sainsbury's Cafe	158	0.5
Soho Coffee	453	1.4
Starbucks	1,784	5.4
Tesco Cafe	53	0.2
The Cornish Bakery	25	0.1
Thomas the Baker	45	0.1
Tim Hortons	201	0.6
**Western Fast Food & Takeaway**	**8043**	**24.4**
Barburrito	129	0.4
Ben & Jerry's	66	0.2
Burger King	108	0.3
Chicken Cottage	66	0.2
Coco Di Mama	1,132	3.4
Crussh	285	0.9
Domino's Pizza	2,436	7.4
FIVE GUYS	74	0.2
KFC	298	0.9
Krispy Kreme	50	0.2
Leon	134	0.4
McDonalds UK	300	0.9
Papa John's	517	1.6
Pieminister	37	0.1
Pizza Hut	598	1.8
PizzaExpress	447	1.4
Pure.	275	0.8
Subway	169	0.5

Taco Bell	328	1.0
Tortilla	27	0.1
Tossed	115	0.3
Waterfield's	28	0.1
Wimpy	424	1.3
**Pubs, Bars, Inns**	**8319**	**25.2**
All Bar One	416	1.3
Brewers Fayre	496	1.5
Brewhouse and Kitchen	185	0.6
Chef and Brewer	402	1.2
Common Room	284	0.9
Cookhouse & Pub	431	1.3
Ember Inns	555	1.7
Farmhouse Inns	470	1.4
Flaming Grill Pub Co.	524	1.6
Greene King	20	0.1
Hungry Horse	555	1.7
Marstons	416	1.3
Nicholson's	397	1.2
ONeills	247	0.7
Revolution Vodka Bars	289	0.9
Sizzling Pubs	745	2.3
Tank and Paddle	172	0.5
Town, Pub & Kitchen	315	1.0
Vintage Inns	497	1.5
Walkabout	177	0.5
Wetherspoon	441	1.3
Yate's	285	0.9
**Restaurants**	**6242**	**18.9**
Ask	220	0.7
Beefeater Grill	470	1.4
Bella Italia	394	1.2
Bills	169	0.5
Browns	577	1.7
Cafe Rouge	304	0.9
GBK	346	1.0
Harvester	813	2.5
Honest Burgers	39	0.1
Loch Fyne	219	0.7
Nandos	269	0.8
Pho	187	0.6
Stonehouse Pizza & Carvery	592	1.8
Table Table	497	1.5

The Real Greek	71	0.2
Toby Carvery	560	1.7
Wagamama	277	0.8
Zizzi	238	0.7
**Sport & Entertainment**	**2155**	**6.5**
Cineworld	524	1.6
ODEON	1,134	3.4
Top Golf	90	0.3
VUE ENTERTAINMENT	407	1.2
**Asian Fast Food**	**569**	**1.7**
Itsu	145	0.4
Wasabi	201	0.6
YO! Sushi	223	0.7
**Total**	**32,981**	**100.0**

Supplementary Table 3. Food groups used in analysis, with descriptions. Adapted from previous work (15).

**Food group**	**Description and examples**
Appetisers and sides	Items that are listed in “appetisers,” “starters,” or “sides” menu sections. Also includes items or small dishes served to complement the main course, for example a chicken skewer “add on” to a burger or meal, or a “side of vegetables/rice/beans/fruit/etc.”
Baked goods	Food items prepared by baking. Examples include bread, cakes, cookies, and pastries.
Beverages	Menu items intended for drinking, including both non-alcoholic and alcoholic options. Examples include water, tea, coffee, juice, and soft drinks.
Burgers	All items described on menus as burgers. Examples include burger, hamburger, chicken burger, veggie burger.
Desserts	All sweets intended to be served as desserts. Also includes baked goods served as desserts (e.g. cakes) and candy. Other examples include cookies and ice cream.
Fried potatoes	Potatoes that have been cooked by frying, often in oil. Examples include French fries, sweet potato fries, potato chips, and hash browns.
Mains	The primary or most substantial item in a meal, usually featuring a significant source of protein. Examples include steak, chicken curry, grilled fish, chicken nuggets.
Pizza	A dish consisting of a flatbread base typically topped with sauce, cheese, and various toppings. Includes items listed as pizzas or flatbreads.
Salads	Dishes composed primarily of raw or cooked vegetables, often mixed with other ingredients and served with a dressing. Examples include garden salads, side salads, or Caesar salads.
Sandwiches	Foods consisting of one or more types of filling, such as meat, cheese, or vegetables, placed between slices of bread or rolls. Examples include ham sandwiches, subs, and wraps.
Soups	Liquid dishes typically made by simmering ingredients like meat, vegetables, and legumes in broth or water. Examples include chicken noodle soup, tomato soup, and minestrone.
Toppings and ingredients	Items added to enhance the flavour of a dish, including condiments, garnishes, and additional components. Examples include cheese, croutons, sauces, dressings. Also includes beverage toppings such as whipped cream added to coffee.

Supplementary Table 4. Mean kcal content of all items from all available MenuTracker chains, by food group and by food business type

	Unadjusted Model			Fully Adjusted Model		
	**Pre-Policy (n=90)**	**Post-Policy All chains (n=104)**	**Difference**	**Pre-Policy (n=90)**	**Post-Policy All chains (n=104)**	**Difference**
	kcal (95% CI)	kcal (95% CI)	kcal (95% CI)	kcal (95% CI)	kcal (95% CI)	kcal (95% CI)
Overall	455 (371 to 539)	439 (355 to 523)	-16 (-25.4 to -6.6)	458 (412 to 505)	450 (404 to 497)	-8 (-8 to 0.2)
**Food Group**	**Pre-Policy**	**Post-Policy All chains**	**Difference**	**Pre-Policy**	**Post-Policy All chains**	**Difference**
Appetisers and sides	336 (237 to 435)	323 (224 to 422)	-13 (-32 to 6)	299 (250 to 348)	290 (242 to 338)	-9 (-30 to 12)
Baked goods	460 (330 to 590)	471 (340 to 601)	11 (-30 to 52)	377 (319 to 435)	365 (309 to 420)	-12 (-58 to 33)
Beverages	171 (137 to 205)	165 (131 to 198)	-7 (-13 to 0.4)	229 (181 to 278)	189 (141 to 237)	-40 (-57 to -33)
Burgers	806 (622 to 990)	719 (536 to 902)	-87 (-148 to -26)	938 (880 to 996)	856 (800 to 912)	-82 (-129 to -36)
Desserts	436 (377 to 495)	438 (381 to 496)	2 (-27 to 32)	393 (343 to 444)	411 (362 to 460)	18 (-9 to 44)
Fried potatoes	421 (376 to 466)	382 (337 to 428)	-39 (-84 to 7)	420 (355 to 485)	358 (293 to 423)	-62 (-127 to 2.7)
Mains	639 (528 to 749)	631 (521 to 740)	-8 (-35 to 19)	718 (670 to 766)	695 (647 to 742)	-23 (-41 to -5)
Pizza	673 (569 to 778)	697 (593 to 801)	24 (6 to 41)	723 (670 to 775)	745 (693 to 797)	22 (1 to 44)
Salads	366 (306 to 426)	400 (342 to 458)	34 (-15 to 84)	370 (310 to 429)	396 (340 to 451)	26 (-23 to 74)
Sandwiches	554 (464 to 644)	629 (542 to 716)	76 (25 to 127)	570 (517 to 622)	646 (595 to 697)	76 (43 to 108)
Soup	271 (211 to 331)	308 (250 to 367)	37 (-12 to 87)	255 (172 to 338)	370 (294 to 447)	115 (25 to 206)
Toppings and ingredients	82 (63 to 102)	96 (76 to 115)	13 (2 to 24)	92 (40 to 145)	101 (47 to 156)	9 (-29 to 47)
**Chain type**						
Cafes	293 (249 to 338)	295 (251 to 339)	2 (-7 to 10)	407 (333 to 481)	407 (333 to 480)	0 (-17 to 17)
Western fast food	462 (363 to 561)	474 (375 to 573)	12 (-5 to 29)	412 (358 to 466)	454 (400 to 508)	42 (27 to 58)
Pubs, bars, and inns	643 (562 to 724)	599 (518 to 679)	-45 (-70 to -19)	580 (521 to 639)	529 (470 to 587)	-51 (-68 to -35)
Restaurants	478	434	-44	474	453	-22

	(438 to 519)	(395 to 473)	(-69 to -20)	(409 to 539)	(388 to 517)	(-41 to -2)
Sport & Entertainment	358<br>(98 to 617)	346<br>(87 to 605)	-12<br>(-34 to 10)	484<br>(347 to 622)	437<br>(301 to 573)	-47<br>(-78 to -16)
Asian fast food	414<br>(251 to 577)	457<br>(295 to 618)	43<br>(-9 to 94)	353<br>(192 to 514)	389<br>(230 to 548)	36<br>(-23 to 95)
